# Differential Expression of miRNAs in Amyotrophic Lateral Sclerosis Patients

**DOI:** 10.1007/s12035-023-03520-7

**Published:** 2023-08-02

**Authors:** Bruno Costa Gomes, Nuno Peixinho, Rita Pisco, Marta Gromicho, Ana Catarina Pronto-Laborinho, José Rueff, Mamede de Carvalho, António Sebastião Rodrigues

**Affiliations:** 1 Instituto de Fisiologia, Instituto de Medicina Molecular, Faculdade de Medicina, Universidade de Lisboa, Lisboa, Portugal; 2 ToxOmics, NOVA Medical School, Faculdade de Ciências Médicas, NMS, FCM, Universidade NOVA de Lisboa, Lisboa, Portugal; 3 Department of Neurosciences and Mental Health, Hospital de Santa Maria CHULN, Lisboa, Portugal

**Keywords:** MicroRNAs, Amyotrophic lateral sclerosis, Biomarkers, Epigenetics

## Abstract

Amyotrophic lateral sclerosis (ALS) is a progressive motor neuron disease that affects nerve cells in the brain and spinal cord, causing loss of muscle control, muscle atrophy and in later stages, death. Diagnosis has an average delay of 1 year after symptoms onset, which impairs early management. The identification of a specific disease biomarker could help decrease the diagnostic delay. MicroRNA (miRNA) expression levels have been proposed as ALS biomarkers, and altered function has been reported in ALS pathogenesis. The aim of this study was to assess the differential expression of plasma miRNAs in ALS patients and two control populations (healthy controls and ALS-mimic disorders). For that, 16 samples from each group were pooled, and then 1008 miRNAs were assessed through reverse transcription-quantitative polymerase chain reaction (RT-qPCR). From these, ten candidate miRNAs were selected and validated in 35 ALS patients, 16 ALS-mimic disorders controls and 15 healthy controls. We also assessed the same miRNAs in two different time points of disease progression. Although we were unable to determine a miRNA signature to use as disease or condition marker, we found that miR-7-2-3p, miR-26a-1-3p, miR-224-5p and miR-206 are good study candidates to understand the pathophysiology of ALS.

**Supplementary Information:**

The online version contains supplementary material available at 10.1007/s12035-023-03520-7.

## Introduction

Amyotrophic lateral sclerosis (ALS) is a motor neuron disease characterised by a progressive degeneration of upper and lower motor neurons [1, 2]. Death usually occurs 3 to 5 years after disease onset, frequently from respiratory complications due to attainment of respiratory muscle [3]. ALS is the most common motor neuron disease in adults, with a global incidence of 0.42 to 2.76 cases per 100,000 person-years and the prevalence varies from 1.57 to 9.62 cases per 100,000 population [4]. Sporadic ALS cases (sALS) account for 90 to 95% of all ALS cases, and about 10% of these have mutations in the familial ALS genes identified thus far (*C9orf71*, *SOD1*, *TARDBP* and *FUS*) [5–7]. The causative pathogenic mechanisms in sALS remain unclear, and besides genetic factors, different epigenetic mechanisms may also contribute to ALS, including DNA methylation, MicroRNAs (miRNAs) expression and regulation and histone modification [8].

A definitive diagnosis of ALS is frequently delayed due in part to heterogeneous phenotypic manifestations and varying speed of progression. Thus, early detection and treatment approaches are crucial for a better outcome [2]. In the last decade, many efforts have been made to find a reliable and non-invasive circulating biomarker for a quick and accurate diagnosis and prognosis [2, 9]. Several biomarkers of different nature and objective are known, namely, biomarkers of exposure, of susceptibility, of effect and of disease. Biomarkers of disease can act as surrogates of clinical endpoints intended to predict the outcome and prognosis, being thus potentially useful in screening and diagnosis and monitoring of disease progression [10].

Accordingly, small non-coding RNAs like miRNAs have been studied as possible biomarkers for several diseases like cancer [11, 12], but also neurodegenerative diseases, including ALS [6, 9, 13–15]. miRNAs are post-transcriptional gene expression regulators with approximately 17 to 25 nucleotides and act by binding to the 3′ untranslated region (UTR) of a target mRNA. One miRNA can regulate several mRNAs, making them important regulators of cellular homeostasis [16]. Due to their biological features, miRNAs are highly stable in several tissues or matrices and are present in both intracellular and extracellular environments [17]. In fact, miRNAs are resistant to extreme conditions of pH and temperature and are resistant to RNase degradation when encapsulated in vesicles or bound to RNA binding proteins. In ALS, cerebrospinal fluid (CSF) has been widely used for biomarker discovery despite the painful process in its extraction and the low amount of volume obtained [9]. Instead, plasma is a more suitable source of biomarkers since sample collection is less invasive and easily obtained [18–20]. Many de-regulated circulating miRNAs have been found in ALS patients and at different disease stages seemingly to be associated with different miRNA profiles [21–23]. However, the potential of miRNA biomarkers for ALS prognosis and as an indicator of disease progression is still not fully explored. In fact, the use of miRNAs as biomarkers for diagnosis and clinical management of patients is still in an early stage of development. Thus, the aim of the present study was to evaluate the usefulness and accuracy of plasma miRNAs levels as putative biomarkers of disease in ALS patients. We here describe the study of three different pooled sampled cohorts, ALS patients, a population of patients with other neuromuscular disorders used as ALS-mimic control and a neurologically normal cohort as a healthy control. We assessed the differential expression of circulating miRNAs in plasma through RT-qPCR in the three populations. Next, we independently validated the most significant results obtained with the pooled samples in 35 ALS patients, 16 ALS-mimic disorders and 15 healthy controls. Additionally, we performed a longitudinal assessment of the same miRNAs in different time points, to evaluate if the miRNAs expression varies along time and disease progression. In Supplementary Fig. 1, we show a flowchart of the number of samples used and which miRNAs were considered candidates for future studies.

## Methods

### Subjects’ Recruitment and Plasma Isolation

Subjects were recruited from the ALS clinic at Department of Neurosciences and Mental Health, Centro Hospitalar Universitário de Lisboa-Norte (CHULN), Lisbon, Portugal. ALS patients were diagnosed according to the Gold Coast criteria [24]. Of an initial number of 116 ALS patients, 35 were selected according to their disease characteristics in order to be as much as possible representative of the disease and its heterogeneity (e.g., balanced number of spinal and bulbar onset and gender, diverse age at onset and progression rate) (Table 1). Patients with infectious diseases or auto-immune diseases (very few), with familial history of ALS, and not Caucasian, were excluded from the selection. None of the patients had a clinical history of cancer diagnosis. All ALS patients were confirmed to be negative for the C9orf72 hexanucleotide repeat expansion (HRE) through genetic screening performed as previously described [25]. The functional rate of change (ΔFS) at sampling was calculated with the Revised ALSFRS (ALSFRS-R) scale [26] as follows: (48-total ALSFRS-R at sampling)/duration in months from onset to sampling. Scores above 0.5 were considered average or fast progression (AF) while the ones under that score were considered slow progressors (S) [27]. Forced vital capacity (FVC) was measured on the days of blood sampling, being part of the patients’ evaluation. The threshold of 80% of the predictive value was used as the lower limit of normal. Of the 35 ALS patients, 23 had a second blood sampling during follow-up, and of these, 16 had a third blood withdrawal. These repeated blood collections allowed us to follow a longitudinal assessment of the miRNAs’ signatures. The ALS-mimic disease controls (*n*=16) were subjects referred with suspected ALS, in whom other diagnoses were made, e.g.: 50% had neuropathies, such as sensory neuropathy, polyneuropathy and chronic inflammatory demyelinating polyneuropathy (CIDP); 40% had myasthenia gravis, and the remaining 10% had myopathies, spinal cord or root lesion. The control cohort (*n*=15) was formed by healthy subjects working at our institutions, matched for age and gender.

**Table 1:** Characterisation of the studied population (*SD*, standard deviation; *n.a*., not applicable)

	ALS	Healthy controls	ALS-mimic controls
Population (*N*)	35	15	16
Average age (SD)–years	58.25 (10.81)	53.84 (12.12)	61.16 (12.17)
Median age–years	59.32	52.00	64.55
Gender (*N*)			
Male	18	9	9
Female	17	6	7
Site of onset (*N*)			
Spinal	26	n.a.	n.a.
Bulbar	9	n.a.	n.a.
Motor neuron predominance (*N*)			
Upper motor neuron	11	n.a.	n.a.
Lower motor neuron	23	n.a.	n.a.
Both	1	n.a.	n.a.
Progression (*N*)			
Slow (ΔFS < 0.5)	20	n.a.	n.a.
Average or fast (ΔFS > 0.5)	15	n.a.	n.a.
% Forced vital capacity (%FVC)			
Normal (≥80%)	23	n.a.	n.a.
Abnormal (<80%)	7	n.a.	n.a.

Whole venous blood was collected into vacutainer K3EDTA tubes, and plasma was immediately isolated by centrifugation (2000 *g* for 10 min, 4 °C) and stored at −80 °C until use. ALS patients, neurologically normal controls and ALS-mimic were gender and age-matched (*p* > 0.1).

This project was approved by the Ethics Committee of Centro Académico de Medicina de Lisboa (CAML) (ref. no. 94/19). The study conformed to the standards defined in the latest revision of the Declaration of Helsinki. All patients signed a written informed consent prior to inclusion into the study. Databases were anonymised and properly treated to safeguard privacy.

### RNA Purification and Quantification

Total RNA, including miRNA fraction, was performed according to the protocol of miRNeasy® Serum/Plasma Advanced Kit (Qiagen - Cat. No. 217204). Briefly, 200 μl plasma was transferred into a 2-ml microcentrifuge tube. Next, 60 μl of Buffer RPL was added to each sample and incubated for 3 min at room temperature (RT). Then, 20 μl of Buffer RPP was added, and a second incubation at RT for 3 min was done. The mixture was then centrifuged at 1200*g* for 3 min. The supernatant was transferred to another microcentrifuge tube, and 1 volume of isopropanol was added. The mixture was then transferred to a Rneasy UCP MinElute column and centrifuged at 8000*g* for 15 s. The supernatant was discarded, and 700 μl of Buffer RWT was pipetted into the column and centrifuged at 8000*g* for 15 s. The supernatant was discarded, 500 μl of Buffer RPE was pipetted into the column, again centrifuged at 8000*g* for 15 s and the supernatant was discarded. At last, 500 μl of 80% ethanol was added into the column and centrifuged at 8000*g* for 2 min. The Rneasy UCP MinElute column was placed in a new 2-ml collection tube and centrifuged at full speed for 5 min to dry the membrane. RNA was eluted from the column in 20 μl of Rnase-free water, incubated 1 min and centrifuged for 1 min at full speed to elute the RNA. Total RNA samples were then stored at −80 °C until further use. Total RNA was quantified using Nanodrop® 2000 spectrophotometer (Thermo Fisher).

### Samples Pooling

In an initial analysis, a pooled sample analysis was performed to select putative miRNA candidates to study in all patients. Thereby, 16 ALS patient samples, representative of the disease and its heterogeneity, were selected: 8 male and 8 female samples; their mean age at study entry was 58.3 years (SD: 11.9); mean age at onset was 56.6 years (SD: 12.1); 8 presented spinal onset form and 8 present bulbar onset form; the mean disease duration at study entry was 20.5 months (SD: 17.5); and mean rate of functional decay was 0.63 (SD: 0.42). Then, 2 μl of total RNA from each sample was used to make the corresponding pool. Following this mixture, the three pools had approximately 30 μl each, which were then used to convert into cDNA. All samples from the ALS-mimic disorders and healthy controls were used to make the respective pooled group. Comparison between groups was performed with the *χ*2 test for gender and Kruskal-Wallis with Dunn’s test for age.

### miRNAs Detection and Data Analysis of the Pooled Samples

The detection of miRNA expression was performed by quantitative RT-PCR (qRT-PCR), according to the protocol of Human miRNome miScript® miRNA PCR Array (Qiagen - Cat. No. 331222) in two steps: First, total RNA was converted into cDNA by reverse transcription reactions using miScript II RT Kit (Qiagen) and performed according to the manufacturers’ protocol. This array allows the detection and quantification of 1008 miRNAs. These are the best characterised miRNAs in the human genome (miRNome) as annotated in miRBase Release 16 [28]. Relative amounts of miRNAs were calculated by using the comparative cycle threshold (Ct) method using the global Ct mean of expressed miRNA as normalisation and compared between groups. Fold change was determined by the 2^-ΔΔCT^. miScript® miRNA PCR array was performed in triplicate. Final data analysis was performed using The GeneGlobe Data Analysis Center, a web resource made available by Qiagen. Only miRNAs with *p* values and corrected *p* values ≤ 0.05 were used. A fold change cut-off of 2.0 was implemented, meaning miRNA were considered upregulated if the fold change values ≥2.0 and down regulated if the values was ≤ −2.0.

### miRNAs Detection and Data Analysis of the Individual and Longitudinal Samples

The miRNAs expression validation in individual samples of ALS patients, ALS-mimic disorders and healthy controls was performed by qRT-PCR using TaqMan miRNA assay kits (Applied Biosystems), following the manufacturers’ protocol. qRT-PCR data was normalised using let-7b-5p and miR-744-5p, and the relative comparisons of miRNAs expression between groups were performed using the 2^-ΔCt^ for individual groups (independent samples). Let-7b-5p and miR-744-5p were used as endogenous controls because in the initial profile of the 1008 miRNAs, these were two miRNAs that showed stable expression in all pooled samples.

### Statistical Analysis

All statistical analysis was carried out in GraphPad Prism 9.0.0 using non-parametric tests: Mann-Whitney test, Friedman test and Kruskal-Wallis’s test (and Dunn’s multiple comparisons). Statistical significance was valued for values of <0.05 with 95% of confidence interval.

### Pathway and Target Enrichment Analysis

miRNAs pathway and target enrichments were done using DIANA mirPath v.3 web server [29]. This web server retrieved miRNAs target genes using DIANA-TarBase v7.0 [30], and pathway enrichment was performed according to the Kyoto Encyclopedia of Genes and Genomes (KEGG) pathways [31, 32]. The *p* value threshold for enrichment was of 0.05, and an FDR correction was performed in all analysis.

## Results

### Pooled Sample Construction

Sixteen samples from ALS patients’ group and all ALS-mimic disorders and healthy control group samples were selected to create pools. The patients for each group were selected with similar average age (ALS patients was 58.3 ±11.92, healthy controls 58.18 ±12.55 and ALS-mimic disorders 57.42 ±13.74, with a *p* value of 0.73) and the same ratio of males and females (ALS patients 8/8, healthy controls 7/8 and ALS-mimic disorders 9/7, with a *p* value of 0.86).

The clinical characterisation of the patients within this study can be seen in Table 1.

### Differentially Expressed miRNAs Using Pooled Samples

**Fig. 1:**
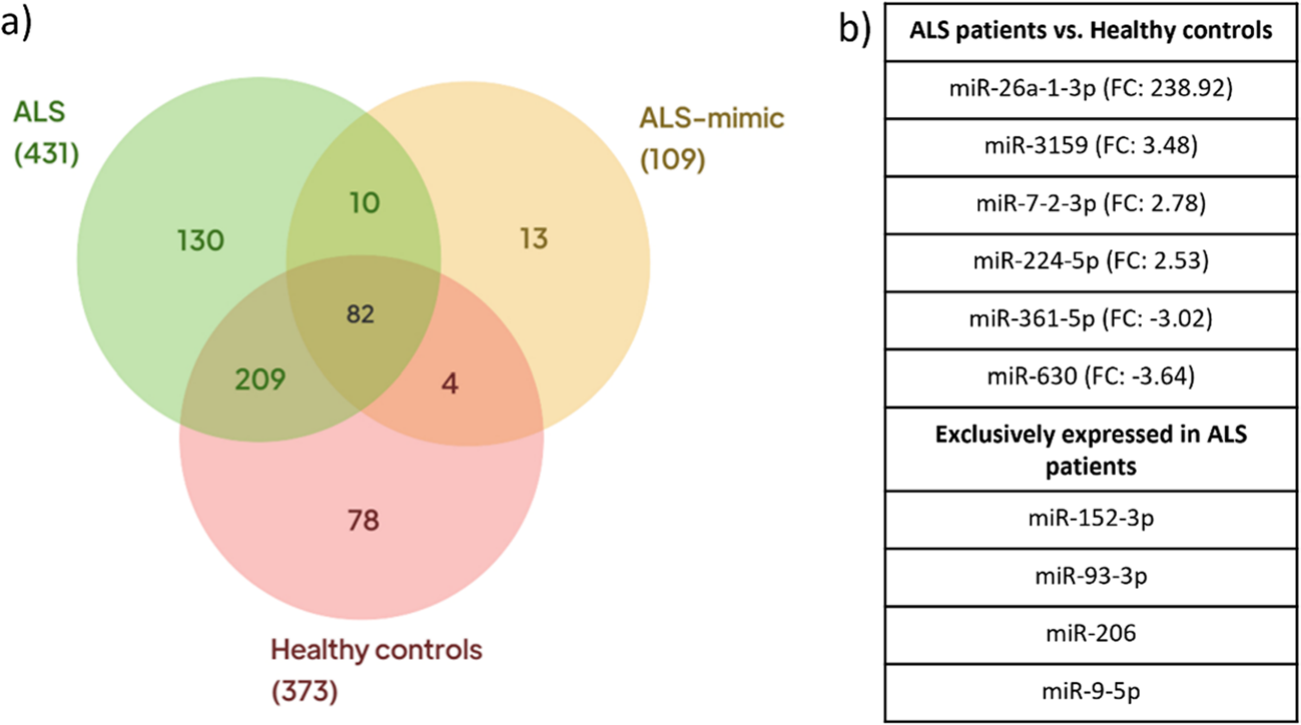
Venn diagram showing the miRNAs expression distribution in ALS, ALS-mimic and healthy controls populations (**a**) and miRNAs selected for individual analysis (**b**). FC, fold change

Of the 1008 miRNAs quantified, 431 were detected in the ALS patients’ pool, 109 in ALS mimic disorders’ pool and 373 in healthy controls (Fig. 1). Of the detected miRNAs, 209 were simultaneously detected in ALS patients and healthy controls, of which six were significantly differently expressed (*p* value < 0.05) and fold change values ≥2.0 or ≤ −2.0. Specifically, miR-26a-1-3p (fold change: 238.92), miR-361-5p (fold change: −3.02), miR-224-5p (fold change: 2.53), miR-7-2-3p (fold change: 2.78), miR-3159 (fold change: 3.48) and miR-630 (fold change: −3.64). Thus, these miRNAs were selected to perform individual and longitudinal analysis as putative relevant disease modulators of ALS. miR-152-3p, miR-93-3p, miR-206 and miR-9-5p were detected among the ALS patients exclusively expressed miRNAs, thus, were also selected for further analysis. These miRNAs were selected using The GeneGlobe Data Analysis Center and were in the top 5 expressed miRNAs in ALS patients.

### Individual and Longitudinal miRNA Analysis

**Fig. 2:**
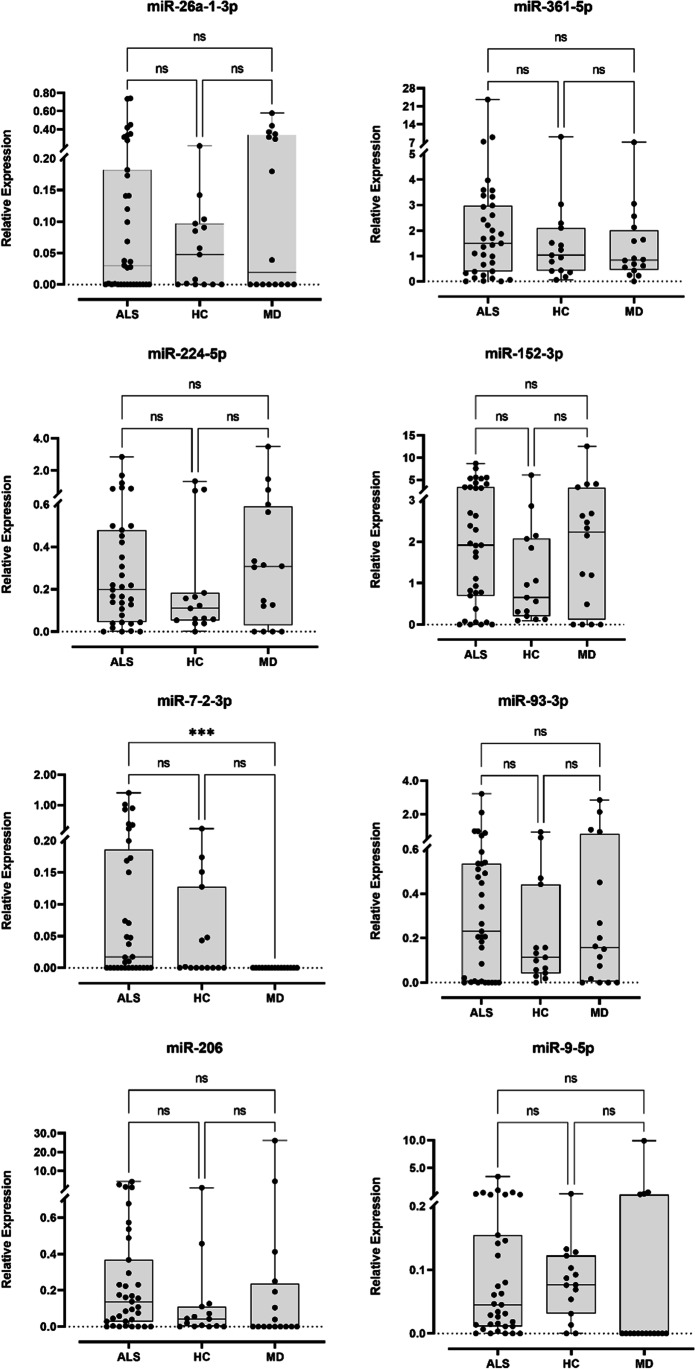
Box and whisker plots of relative expression of the 8 miRNAs detected in ALS (*N* = 35), ALS-mimic disorders (*N* = 16) and healthy controls (*N* = 15). Dots represent mean relative expression values of each sample. Statistical significance calculated using Kruskal-Wallis test and Dunn’s multiple comparisons test. ***p value < 0.001

The set of ten miRNAs identified through the pooled analysis was then validated in individual and longitudinal samples through RT-qPCR. Two miRNAs, miR-3159 and miR-630 were not detected in any sample. From the remaining eight miRNAs, no statistical differences were observed between the different populations (Fig. 2). In fact, miR-152-3p, miR-93-3p, miR-206 and miR-9-5p, which in the pooled samples were only detected in ALS patients, were found expressed in the three populations. However, not all samples from these populations showed expression of these miRNAs. Regarding miR-26a-1-3p, miR-361-5p, miR-224-5p, miR-7-2-3p, miR-3159 and miR-630, that were selected because they showed statistically significant differences between ALS and healthy control populations in pooled samples, showed no differences when individually analysed. Only miR-7-2-3p showed statistically significant differences (*p* value = 0.0004) between ALS patients and ALS-mimic disorders patients (Fig. 2). In fact, no ALS mimic disorder sample expressed miR-7-2-3p.

**Fig. 3:**
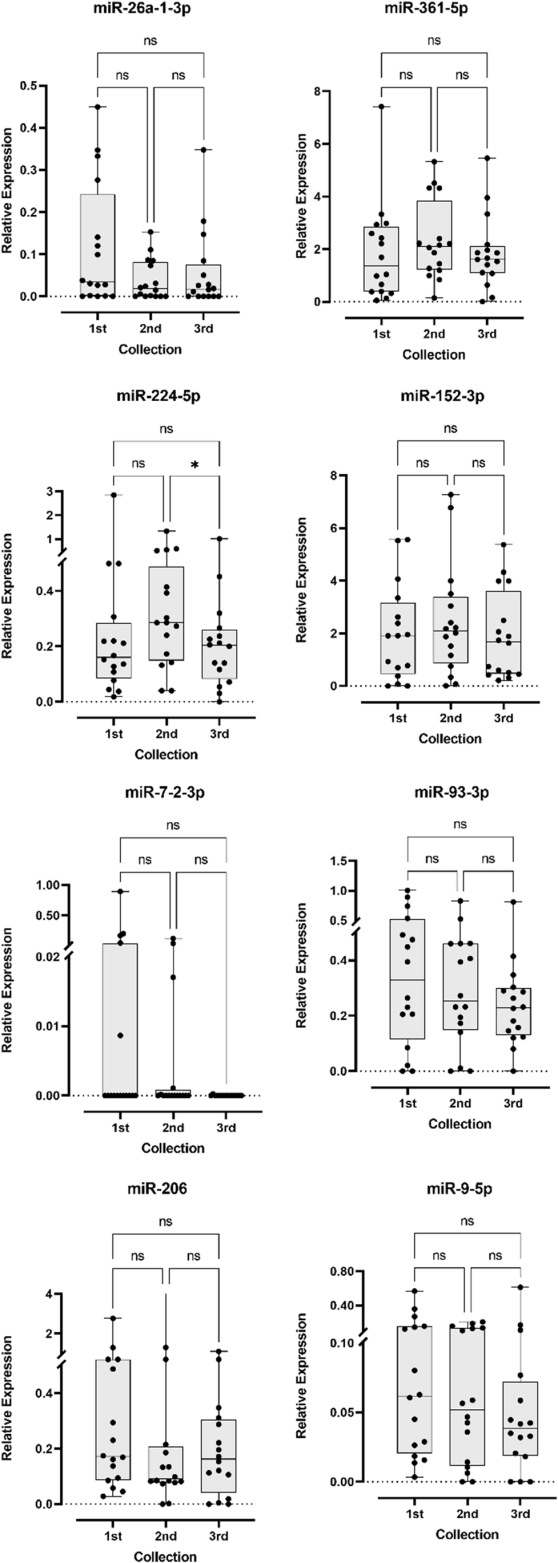
Box and whisker plots of relative expression of the 8 miRNAs detected in ALS longitudinal samples. Dots represent mean relative expression values of each sample. Statistical significance calculated using Kruskal-Wallis test and Dunn’s multiple comparisons test. *p value < 0.05

Regarding longitudinal analysis, 16 of the 35 of our ALS patients had their blood collected at three different time moments, with a median interval of 5.36 months. Only miR-224-5p showed significant differences (*p* value = 0.024) between samplings (Fig. 3), precisely, between the second and the third. This miRNA seems to have higher expression levels at the second collection time, even though the difference in relative expression is not statistically significant between the first and the second collection. Globally, all miRNAs showed an heterogenous relative expression between collections. In fact, analysing sample by sample, we can observe a high miRNA expression variation between collections.

### miRNAs Relative Expression and Clinical Data Integration

Given the heterogeneity and complexity of ALS, we grouped our 35 ALS patients according to selected clinical traits, such as the site of onset, the rate of disease progression, the forced vital capacity status at first collection and motor neuron predominance. We also stratified our data according to gender. Comparison of relative miRNA expression between each of these groups is summarised in Table 2.

**Table 2:** Comparison of relative miRNA expression between groups of ALS patients with clinically distinct characteristics. Values are expressed as median and inter quartile range (Q1–Q3). Statistical significance calculated using Mann-Whitney test

miRNA	Spinal vs bulbar	*p* value	ΔFS<0.5 vs ΔFS >0.5	*p* value	FVC ≥80% vs FVC <80%	*p* value	UMN vs LMN	*p* value	Female vs male	*p* value
miR-26a-1-3p	0.084 (0.001–0.318) 0.000 (0.000–0.276)	0.009	0.034 (0.000–0.250) 0.028 (0.000–0.182)	0.600	0.036 (0.000–0.276) 0.030 (0.000–0.141)	0.522	0.099 (0.099–0.347) 0.028 (0.000–0.141)	0.407	0.036 (0.000–0.227) 0.029 (0.000–0.206)	0.854
miR-361-5p	1.780 (0.717–3.080) 0.237 (0.008–2.500)	0.055	1.070 (0.258–2.850) 1.680 (0.732–3.580)	0.433	1.860 (0.384–2.980) 0.982 (0.016–1.430)	0.131	1.860 (0.982–3.580) 1.490 (0.384–2.930)	0.462	1.43 (0.310–3.003) 1.688 (0.377–3.068)	0.800
miR-224-5p	0.204 (0.122–0.458) 0.039 (0.000–0.684)	0.288	0.132 (0.040–0.283) 0.351 (0.197–0.870)	0.023	0.220 (0.127–0.499) 0.107 (0.003–0.197)	0.056	0.220 (0.043–0.870) 0.197 (0.079–0.451)	0.744	0.265 (0.090–0.869) 0.159 (0.042–0.429)	0.206
miR-152-3p	1.950 (0.774–4.360) 1.63 (0.027–3.030)	0.239	2.110 (0.458–4.960) 1.750 (0.767–3.350)	0.730	2.380 (0.699–4.330) 0.929 (0.055–1.910)	0.133	2.690 (1.910–5.260) 1.110 (0.378–3.350)	0.106	1.746 (0.228–3.712) 2.114 (0.750–3.595)	0.883
miR-7-2-3p	0.059 (0.000–0.248) 0.000 (0.000–0.000)	0.002	0.027 (0.000–0.145) 0.000 (0.000–0.339)	0.904	0.010 (0.000–0.074) 0.150 (0.000–0.200)	0.422	0.049 (0.000–0.339) 0.017 (0.000–0.150)	0.714	0.017 (0.000–1.398) 0.009 (0.000–0.071)	0.348
miR-93-3p	0.305 (0.069–0.591) 0.006 (0.000–0.492)	0.126	0.250 (0.009–0.525) 0.207 (0.000–0.588)	0.987	0.207 (0.021–0.588) 0.231 (0.000–0.510)	0.477	0.536 (0.006–0.742) 0.207 (0.000–0.492)	0.160	0.207 (0.001–0.565) 0.305 (0.069–0.535)	0.682
miR-206	0.165 (0.071–0.500) 0.001 (0.000–0.135)	0.014	0.200 (0.048–0.524) 0.085 (0.000–0.156)	0.046	0.169 (0.040–0.536) 0.045 (0.000–0.223)	0.118	0.075 (0.004–0.231) 0.169 (0.028–0.536)	0.325	0.075 (0.001–0.165) 0.331 (0.041–0.830)	0.008
miR-9-5p	0.054 (0.014–0.148) 0.031 (0.000–0.635)	0.611	0.067 (0.014–0.239) 0.034 (0.000–0.080)	0.297	0.063 (0.016–0.214) 0.003 (0.000–0.123)	0.055	0.045 (0.146–0.214) 0.046 (0.011–0.155)	0.643	0.031 (0.005–0.110) 0.070 (0.018–0.290)	0.123

**Fig. 4:**
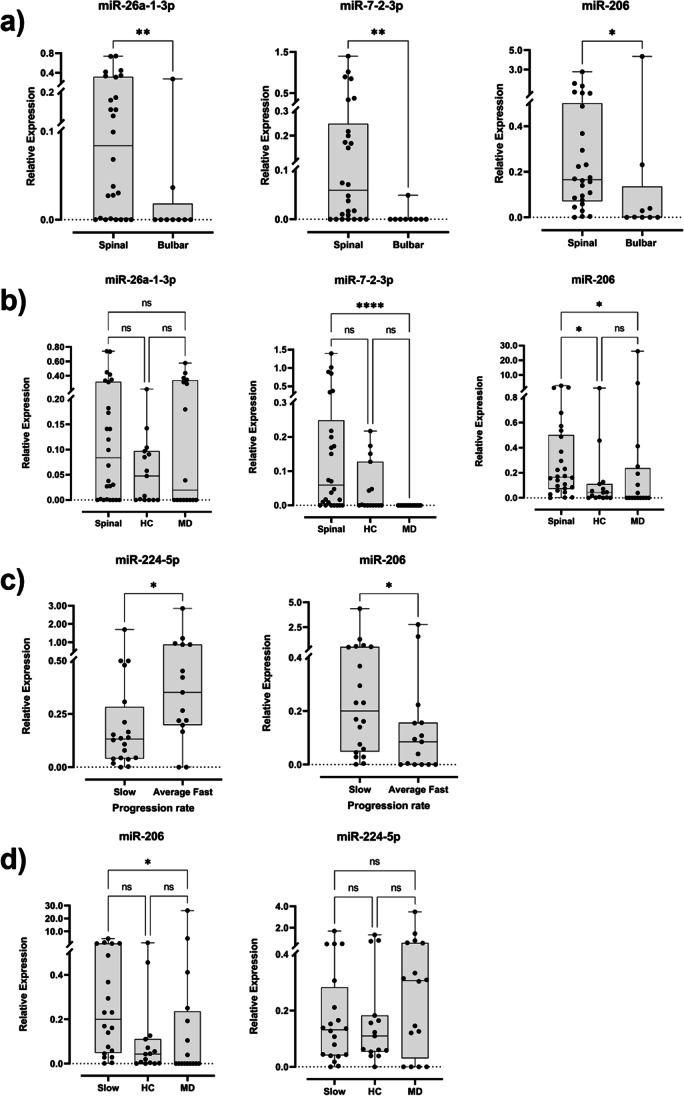
Box and whisker plots of relative expression of the statistically differently expressed miRNAs detected in **a** spinal (*N* = 26) and bulbar (*N* = 9) onset patients, **b** spinal onset patients and control groups, where HC and MD stand for healthy and ALS-mimic diseases control groups respectively, **c** progression rate (*N* (slow) = 20, *N* (average fast) = 15) and **d** slow progression patients and control groups. Dots represent mean relative expression values of each sample. Statistical significance calculated using Kruskal-Wallis test and Dunn’s multiple comparisons test. *p value < 0.05

When comparing the relative expression of each miRNA between the site of onset, three miRNAs (miR-26a-1-3p, miR-7-2-3p and miR-206) showed statistically significant differences (*p* values = 0.009, 0.002 and 0.014, respectively). In fact, all are overexpressed in the spinal onset subgroup when compared with the bulbar onset subgroup (Fig. 4a). Due to the fact that from the 16 patients of the longitudinal analysis, 14 were patients with spinal onset, we performed a longitudinal analysis with these 14 patients (Supplementary Fig. 1). Here, miR-224-5p remains statistically significantly overexpressed from the second collection to the third (*p* value = 0.014), and miR-7-2-3p appears differentially expressed from the first collection to the second and third collections, but only statistically significant when compared with the last (*p* value = 0.025; supplementary Fig. 2). The same trend was seen in the general longitudinal analysis but without any statistical significance. We also compared these three differently expressed miRNAs in spinal onset stratification with control populations, and we found that miR-26a-1-3p has no statistical differences between populations, and miR-7-2-3p is overexpressed in the spinal subgroup when compared with the ALS-mimic diseases group. miR-206 expression was significantly increased when compared to both control groups (*p* value spinal vs healthy controls = 0.044, *p* value spinal vs ALS mimic disorders = 0.022, Fig. 4b).

Concerning the rate of disease progression in our 35 ALS patients, only miR-224-5p and miR-206 were found statistically differentially expressed (*p* values = 0.023 and 0.046, respectively). miR-224-5p was found significantly less expressed in the slow progression subgroup while miR-206 is significantly higher on the same group (Fig. 4c). From the 16 longitudinal samples, 12 are from slow progression patients, then, the same approach as before was done and analysed their expression when comparing different collections through time. Here, only miR-7-2-3p expression was found significantly different between collections (*p* value = 0.024), with a consistent longitudinal decrease in expression (Supplementary Fig. 3). Since we only obtained statistical significance for miR-224-5p and miR-206 progression rate, we compared the relative expression of these two miRNAs with both controls. From these, only miR-206 showed a significant difference in expression between the slow progression patients and the ALS-mimic disorders group (*p* value =0.021, Fig. 4d).

Regarding the pulmonary function status at first collection, no differences in the relative expression of studied miRNAs were found when comparing the subgroup of patients with forced vital capacity (FVC)> 80% at first sampling and the subgroup of patients with %FVC ≤ 80%. This analysis was performed with only 30 patients instead of 35, due to the lack of data for the remaining 5.

Considering motor neuron predominance, we found no statistical significance in the miRNAs studied.

When stratifying our three groups by gender and comparing them separately, we only obtained significantly differences in miR-7-2-3p expression between ALS and ALS-mimic disorders groups (Supplementary Figs. 4 and 5). Considering only the ALS group, only miR-206 revealed to be statistically different between female and male (*p* value = 0.008, Supplementary Fig. 6).

### Enrichment Pathway Analysis

**Fig. 5:**
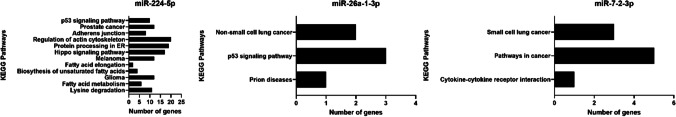
Enriched KEGG pathways of miR-224-5p, miR-26a-1-3p and miR-7-2-3p using mirPath v.3.0 package from DIANA tools. All pathways showed are significantly enriched (*p* value < 0.05)

To better understand the possible pathways and target genes of the relevant miRNAs analysed, we used the mirPath v.3.0 package from DIANA tools [29] to perform a pathway analysis. This data base compares each miRNA target to all Kyoto Encyclopedia of Genes and Genomes (KEEG) pathways. We used DIANA TarBase v.7.0 which provides an enrichment using experimentally validated miRNA-gene interactions. Hsa-miR-224-5p might regulate 12 different pathways while Hsa-miR-26a-1-3p and hsa-miR-7-2-3p might be involved in three pathways each. Among the most enriched pathways by Hsa-miR-224-5p are “Regulation of actin cytoskeleton,” “Protein processing in ER” and “Hippo signalling pathway.” With less genes enriched are lipid-related pathways, precisely, “Fatty acid metabolism,” “Biosynthesis of unsaturated fatty acids” and “Fatty acid elongation” (Fig. 5). The putative targets of Hsa-miR-224-5p are listed in Supplementary Table 1. The enriched pathways of Hsa-miR-26a-1-3p were “Prion diseases,” “p53 signalling pathway” and “Non-small-cell lung cancer.” The enriched pathways of hsa-miR-7-2-3p were “Cytokine-cytokine receptor interaction,” “Pathways in cancer” and “Small-cell lung cancer.” The putative targets of both miRNAs can be seen in Supplementary Table 1.

## Discussion

The importance of miRNAs as disease biomarkers has been increasing in the past years, particularly in cancer [11, 33–36], cardiac diseases [37, 38] and several neurodegenerative diseases [39–41], including ALS [42–44]. A global downregulation of miRNAs in motor neurons has been described in ALS patients in comparison with healthy controls and other neurodegenerative patients [7, 45]. From the ten candidate miRNAs studied in individual and longitudinal samples, miR-26a-1-3p, miR-361-5p, miR-224-5p, miR-7-2-3p, miR-3159 and miR-630 were significantly differently expressed between ALS patients and healthy controls; and miR-152-3p, miR-93-3p, miR-206 and miR-9-5p were exclusively expressed in ALS patients. None of these miRNAs was found expressed in ALS-mimic disorders pool, which was the purpose since this way we could discard miRNAs related to neurodegeneration but unspecific to ALS. In the following individual analysis, none of the studied miRNAs was found statistically differently expressed between ALS and healthy control groups, and only one miRNA, miR-7-2-3p, was found differently expressed between ALS and ALS-mimic disorders. In fact, miR-7-2-3p was not detected in any sample of the ALS-mimic disorders samples. Moreover, miR-630 and miR-3159 were not expressed in any sample used in the individual analysis.

Concerning miR-7-2-3p, which is considered a tumour suppressor [46], there is no evidence about the direct association with ALS or neurodegeneration. Nonetheless, our enrichment pathway analysis showed that miR-7-2-3p might regulate *CCND1*, which expresses Cyclin D1 protein (a CDK kinases regulator protein) and, consequently, regulates the G1 to S transition in the cell cycle [47]. A study in an animal model of ALS showed that the upregulation of *CCDN1* and Cyclin D1 activates the Wnt/β-catenin pathway [48]. This activation is associated with glial proliferation in the spinal cord of the animal model, as a protective mechanism for the neurons during the progression of the disease. Since in our data miR-7-2-3p is overexpressed in ALS patients, we can suggest that a downregulation of Cyclin D1 and consequent deactivation of Wnt/β-catenin pathway might increase the disease progression. However, thorough studies need to be done in order to verify this hypothesis. The same result was obtained to the spinal onset subgroup and longitudinal analysis, where the first collection shows higher levels of miR-7-2-3p. As we pointed out before, this miRNA might regulate a gene that has been associated with glia proliferation in the spinal cord of an ALS animal model. Given that glia proliferation in the spinal cord is a histopathologic feature of ALS [1, 49], we propose that miR-7-2-3p is actually overexpressed in the beginning as response to the onset of the disease and consequent motor neuron damage. That would explain the overexpression of this miRNA in the first collection of spinal onset subgroup and slow progression subgroup, and the differences on relative expression between bulbar and spinal subgroups. Bulbar onset patients are usually associated with a more aggressive and faster disease progression [50]. According to our longitudinal analysis, miR-7-2-3p is highly expressed in the beginning of the disease and then completely depleted. Thus, miR-7-2-3p might be a protective factor against neurodegeneration in general, which is triggered with the neurodegeneration onset. When its effect is not able to halt the disease progression and the neurodegeneration in general, it ceases to be expressed at all. Taking into consideration these results, further molecular and functional studies of miR-7-2-3p role in ALS are of interest to understand ALS pathophysiological mechanisms and neurodegeneration.

Regarding miR-26a-1-3p, our general relative expression and longitudinal analysis did not reveal significant differences between groups. However, when we stratified the samples according to the clinical data, miR-26a-1-3p was found overexpressed in the spinal onset subgroup when compared with the bulbar onset subgroup, in which it almost did not express at all (only two samples from a total of nine samples expressed this miRNA). To our knowledge, miR-26a-1-3p is not associated with ALS. Thus, through our enrichment pathway analysis, we found that miR-26a-1-3p might regulate non-small-cell lung cancer, p53 signalling pathway and prion diseases pathways. In this latter, miR-26a-1-3p interacts with *PRNP*; however, there is no evidence of this gene to be involved in ALS. Concerning p53 signalling, this acts in several cell responses to stresses, such as DNA damage, hypoxia and neuronal death [51–55]. Also, some evidence shows that p53 signalling pathway is involved in ALS and neuronal death [54, 56, 57], with some of the genes regulated by miR-26a-1-3p (*IGFBP3*, CCND1 and *CDK6*) being deregulated in the process. It was already shown that TDP-43 depletion results in an upregulation of *CDK6* that might lead to a cell cycle arrest in G1 and consequently apoptosis [58]. According to our data, miR-26a-1-3p is under expressed in bulbar onset; thus, being a putative regulator of *CDK6*, this might contribute to the worse outcome of bulbar onset ALS. Taking into consideration our enrichment pathway analysis and the literature, miR-26a-1-3p might be part of the complex ALS pathophysiology, and it will be worth of further studies.

MiR-224-5p has never been associated with ALS in the literature, and according to our longitudinal analysis, it seems to have a decreased expression level in the later disease stages. Beside of being significantly different expressed between the second and third collections in the general longitudinal analysis, miR-224-5p also shows the same result in the spinal onset longitudinal analysis. Also, this miRNA is under expressed in the slow progression subgroup when compared to the average or fast subgroup. In both longitudinal analysis, we see a decrease in expression levels from the second to the third collections, suggesting a lower expression of miR-224-5p in late disease stages. Even though it was not significant, from the first to the second collection, we see an increase in expression of miR-224-5p, which in turn suggests that this miRNA might have a peak of expression in the mid stages of the disease and then starts to decrease again. Our enrichment pathway analysis showed that miR-224-5p is involved in several pathways. Among others, it is relevant to highlight the putative role in fatty acid metabolism, biosynthesis of unsaturated fatty acids and fatty acid elongation. Some studies showed that in ALS patients, fatty acid metabolism is impaired, and the energy expenditure is increased to a hypermetabolic state [59, 60]. In fact, Steyn and colleagues [60] demonstrated that an increased fatty acid oxidation is correlated with the hypermetabolic state and high energy expenditure. In ALS, there are evidences of altered cytoskeleton dynamics, especially in actin regulators profilins [61]. This family of proteins can bind to monomeric G actin and facilitates the ADP–ATP transformation. Depending on the situation, profilins can promote actin polymerisation or sequester actin molecules [62]. In fact, profilin 1 has been suggested as a player in ALS pathophysiology, contributing to the TDP-43 aggregations due to loss of ability to interact with the stress granules [63], while profilin 2a has a key role in the Rho-kinase (ROCK) pathway. It is known that ROCK inhibition might increase motor neuron survival [64, 65]. Considering the importance of this pathway in ALS and the fact that the enrichment pathway analysis of miR-224-5p revealed a putative role in the regulation of the actin cytoskeleton, further studies of this miRNA in ALS pathophysiology are of utmost importance. One of the putative targets of miR-224-5p is ARHGAP3 or CHN2. This directly inhibits RHO, which is responsible for stimulating ROCK, that in turn inhibits profilins binding activity and actin polymerisation. Thus, miR-224-5p might have an inhibiting effect on ROCK and consequently on profilins. Also, miR-224-5p might regulate *VAV3*, *PAK2* and *PIP4K2B* genes. VAV3 regulates GTPase activity of RAC1 [66], which has been suggested as a player in ALS pathophysiology by regulating actin and microtubule cytoskeleton and in NADPH-dependent membrane oxidase (NOX) [67]. In sum, miR-224-5p seems to play a role in multiple pathways affecting ALS pathophysiology, thus, further studies should be conducted to better understand miR-224-5p role in ALS.

Finally, miR-206 is the most investigated miRNA in ALS context and the one that shows higher potential as a biomarker. In fact, a total of eight studies reported an upregulation of miR-206 in ALS patients’ samples [68–75]. When we clustered the subgroups according to clinical characteristics, miR-206 showed an overexpression in the slow progression subgroup (comparing to the average or fast progression subgroup) and in the spinal onset subgroup (compared with bulbar onset subgroup). Since we have a reasonable number of patients in our spinal onset subgroup (26), and the characteristic site of onset was the one that revealed more significantly different expressed miRNAs, we decided to compare those miRNAs in that subgroup with the control groups. In this comparisons, miR-206 was overexpressed in ALS patients, when comparing to both control groups. We did the same to the slow progression subgroup versus the average or fast progression subgroup. In this second comparisons, miR-206 is also overexpressed in the subgroup when compared to the ALS-mimic group, and it shows a higher relative expression than the heathy control group, even though it is not statistically significant. Our results support data from the studies concerning the expressing pattern of miR-206 in ALS patients. *Dobrowolny* and colleagues highlight the use of this miRNA as a potential prognostic biomarker since it is highly expressed in the early stages of ALS patients with slower progression. The use of miRNA expression levels to stratify patient’s cohort in different subgroups can have great value in clinical practice, allowing the assessment of the efficacy of therapeutic compounds in clinical trials [73]. Two studies reported the upregulation of miR-206 in ALS samples of plasma [70, 76]. Besides finding miR-206 upregulated in skeletal muscle biopsies, de Andrade and colleagues [70] also tested plasma and found that miR-206 was also overexpressed in these samples. Soliman et al. [76] obtained the same result for an Egyptian population. A study performed in an ALS mice model has demonstrated that miR-206 effects are modulated by muscle-derived factors promoting nerve-muscle interactions in response to motor neuro damage [77]. This work preceded other studies, and years later, there was more evidence on miR-206 compensatory effect in ALS. *Bruneteau* and colleagues [69] showed that there is a correlation between muscle reinnervation modulated by miR-206 and disease progression, suggesting a protective effect of this miRNA by regenerating neuromuscular junctions. That study also pointed out that the proportion of reinnervated neuromuscular junctions was higher in long-term survivors of the disease, with a slower and less aggressive progression. Another study [78] reinforced these results showing the upregulation of miR-206 during muscle re-innervation and during ALS disease. It demonstrated, once more, that miR-206 can attenuate ALS progression through regeneration of neuromuscular junctions. Likewise, the study found that miR-206 expression levels vary according to disease progression. In the beginning of slow progressive disease, there is an increase in miR-206 expression before a decline. *De Andrade* and colleagues [70] found the same results analysing samples of plasma and skeletal muscle of ALS patients. Precisely, they found that miR-206 is overexpressed but this pattern was not observed over 6 and 12 months of follow-up. The hypothesis purposed is that miR-206 expression increases in the early stages of the disease as a response to the motor neuron degeneration reaching a plateau and then begins to fall.

Additionally, we performed an analysis of the GWAS catalog [79] and webTWAS database [80], which allow us to understand how genetic basis of complex traits and diseases might be influenced by genetic variants and gene expression. Although these databases do not directly address miRNA-related complex traits and diseases associations, they can provide valuable information about the putative targets of the miRNAs studied. Analysing the genes mentioned before as putative targets of the candidate miRNAs, no direct association with ALS was found. However, some variants found in these genes using GWAS catalog deserve to be mentioned. Specifically, *CCND1* variants rs1944129 and rs2510461 which are associated with lung function according to GWAS catalog, might be relevant to ALS patients with respiratory issues [81]. Also *PRNP* variants rs1799990, rs6107516 and rs6116492, which are associated with Creutzfeldt-Jakob disease and share some symptoms with ALS, and *IGFBP3* variants rs10597497, rs2965072 and rs1542820, which are associated with brain volume and morphology changes, are potentially relevant to ALS [82]. Using the webTWAS database, we did not find any association of these genes expression with ALS.

## Conclusions

In summary, we report the analysis of ten miRNAs expression levels in ALS patients and compared these expression levels with two control populations (healthy controls and ALS-mimic disorders controls). This approach using two populations as controls is a strength of this work to rule out possible miRNAs involved in other neurological disorders than ALS. Although we were unable to determine a miRNA signature to use as disease or condition marker, from the ten miRNAs we highlight miR-7-2-3p, miR-26a-1-3p, miR-224-5p and miR-206 as candidate miRNAs for further functional studies to ascertain their role in ALS pathophysiology. In the future, it will be crucial to broaden our research efforts by looking at a larger sample size that includes a variety of populations. This would give us a deeper understanding of how these identified miRNAs relate to ALS. Future research must also focus on examining the possible therapeutic implications of our results, such as their value in early diagnosis or as targets for new treatment approaches.

### Supplementary Information

ESM 1 Supplementary Fig. 1 Flowchart showing sample size studied and candidate miRNAs selected for future studies. (PNG 154 kb)

ESM 2 Supplementary Fig. 2 Box and whisker plots of relative expression of the statistically differently expressed miRNAs detected in spinal onset patients, considering longitudinal samples. Dots represent mean relative expression values of each sample. Statistical significance calculated using Kruskal-Wallis test and Dunn’s multiple comparisons test. * p value < 0.05. (PNG 217 kb)

ESM 3 Supplementary Fig. 3 Relative expression of the 8 miRNAs in longitudinal samples of slow progression rate patients. Dots represent mean relative expression values of each sample. Statistical significance calculated using Kruskal-Wallis test and Dunn’s multiple comparisons test. * p value < 0.05 (PNG 257 kb)

ESM 4 Supplementary Fig. 4 Box and whisker plots of relative expression of miR-7-2-3p considering only females. ALS – N = 17, HC – N = 6 and MD – N = 7. Dots represent mean relative expression values of each sample. Statistical significance calculated using Kruskal-Wallis test and Dunn’s multiple comparisons test. * p value < 0.05. (PNG 249 kb)

ESM 5 Supplementary Fig. 5 Box and whisker plots of relative expression of miR-7-2-3p considering only males. ALS – N = 17, HC – N = 6 and MD – N = 7. Dots represent mean relative expression values of each sample. Statistical significance calculated using Kruskal-Wallis test and Dunn’s multiple comparisons test. * p value < 0.05. (PNG 246 kb)

ESM 6 Supplementary Fig. 6 Box and whisker plot of relative expression the statistically differently expressed miRNA detected comparing female and male ALS patients. Female – N = 17, Male – N = 18. Dots represent mean relative expression values of each sample. Statistical significance calculated using Mann-Whitney test. ** p value < 0.01. (PNG 290 kb)

ESM 7 Supplementary Table 1 miR-224-5p, miR-26a-1-3p and miR-7-2-3p enriched KEEG pathways and putative targets (XLSX 13.1 kb)

## Data Availability

The datasets generated analysed during the current study are not publicly available but are available from the corresponding author on reasonable request.
