# Supplementary figures and images for: Differential Expression of miRNAs in Amyotrophic Lateral Sclerosis Patients
Source: Mol Neurobiol. 2023 Aug 2;60(12):7104–17. doi: 10.1007/s12035-023-03520-7 (PMC10657797; doi:10.1007/s12035-023-03520-7)

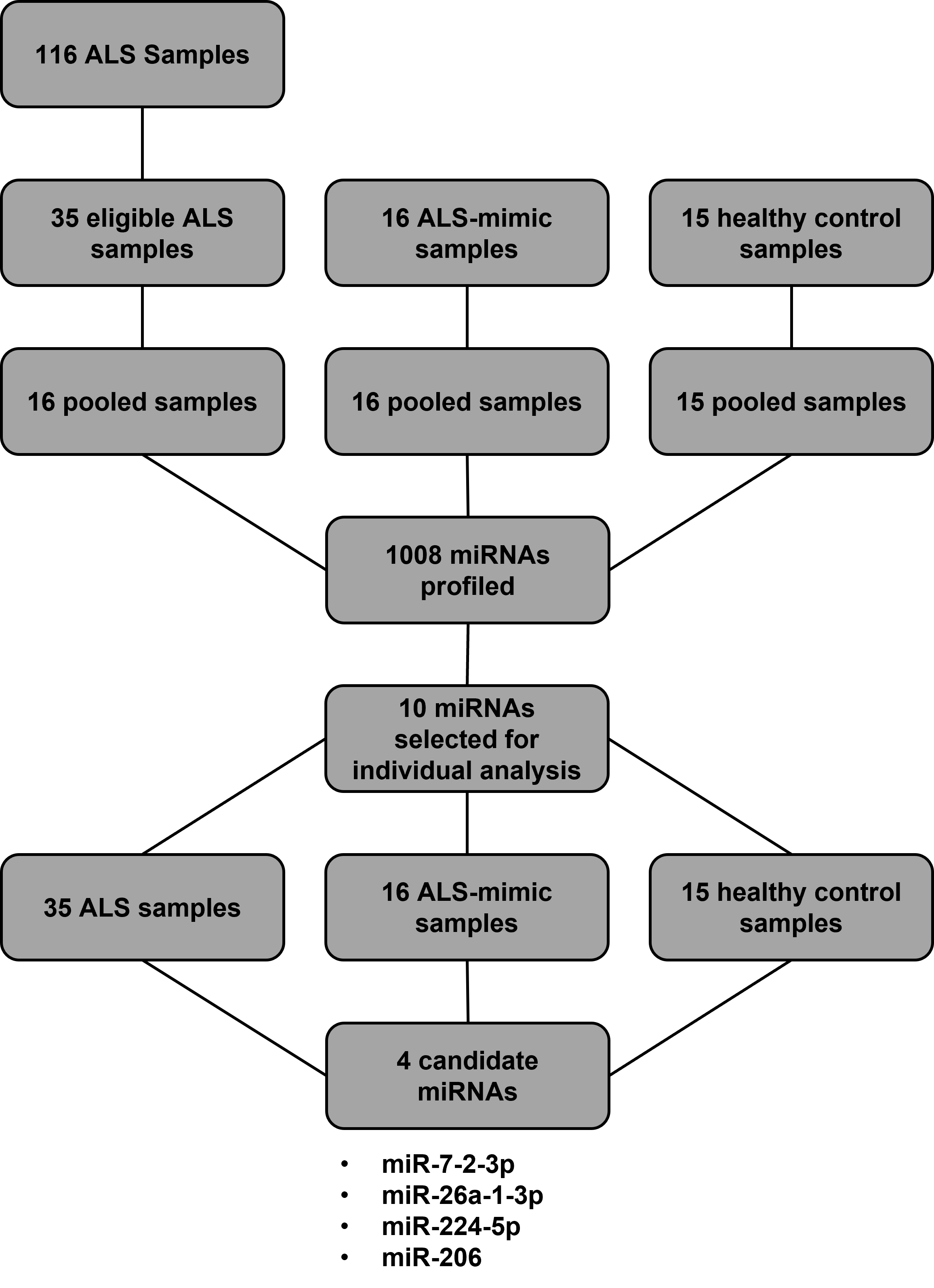

Supplement: Supplementary file 1 — Supplementary Fig. 1 Flowchart showing sample size studied and candidate miRNAs selected for future studies. (PNG 154 kb) [file 12035_2023_3520_MOESM1_ESM.png]

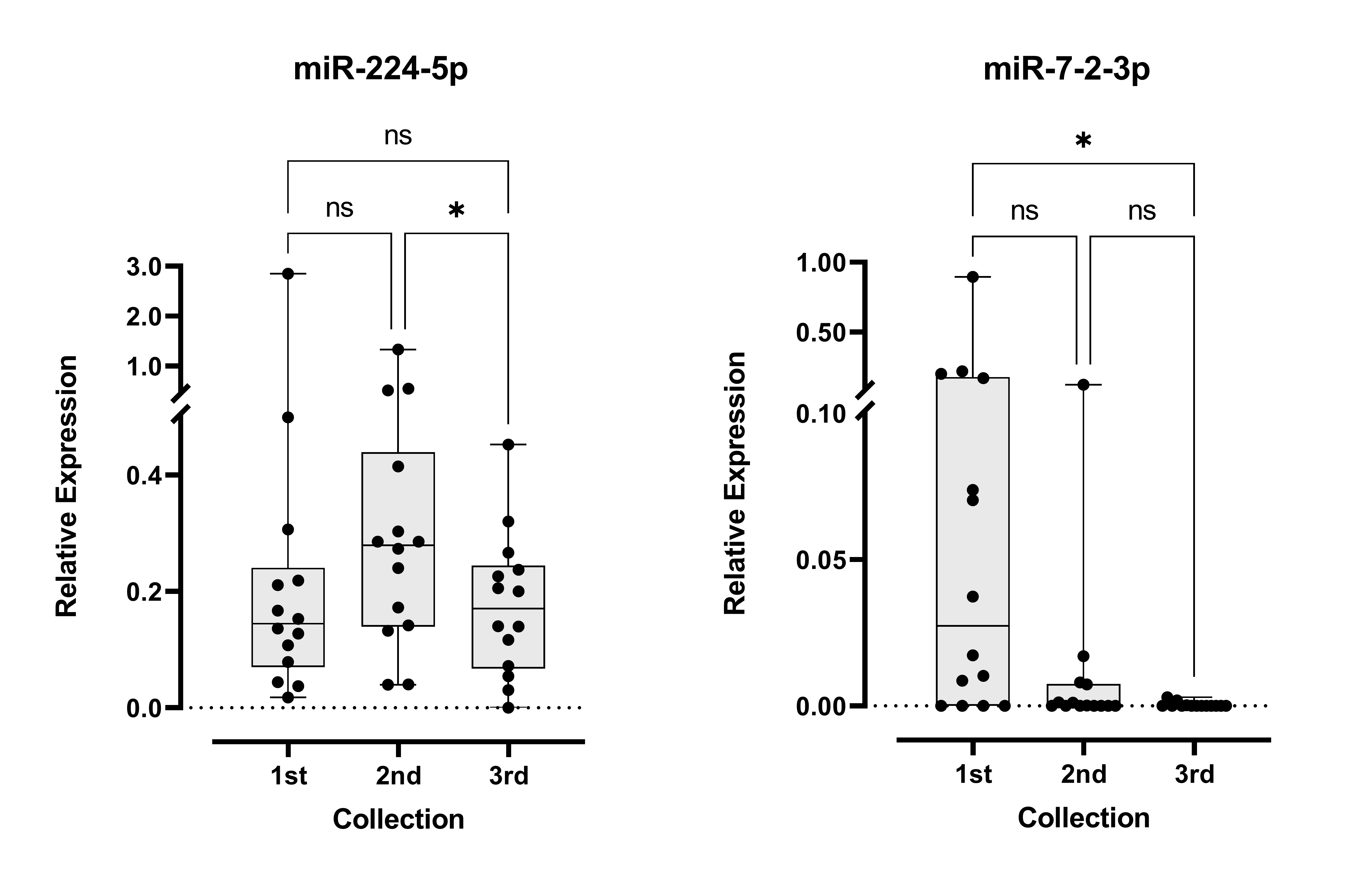

Supplement: Supplementary file 2 — Supplementary Fig. 2 Box and whisker plots of relative expression of the statistically differently expressed miRNAs detected in spinal onset patients, considering longitudinal samples. Dots represent mean relative expression values of each sample. Statistical significance calculated using Kruskal-Wallis test and Dunn’s multiple comparisons test. * p value < 0.05. (PNG 217 kb) [file 12035_2023_3520_MOESM2_ESM.png]

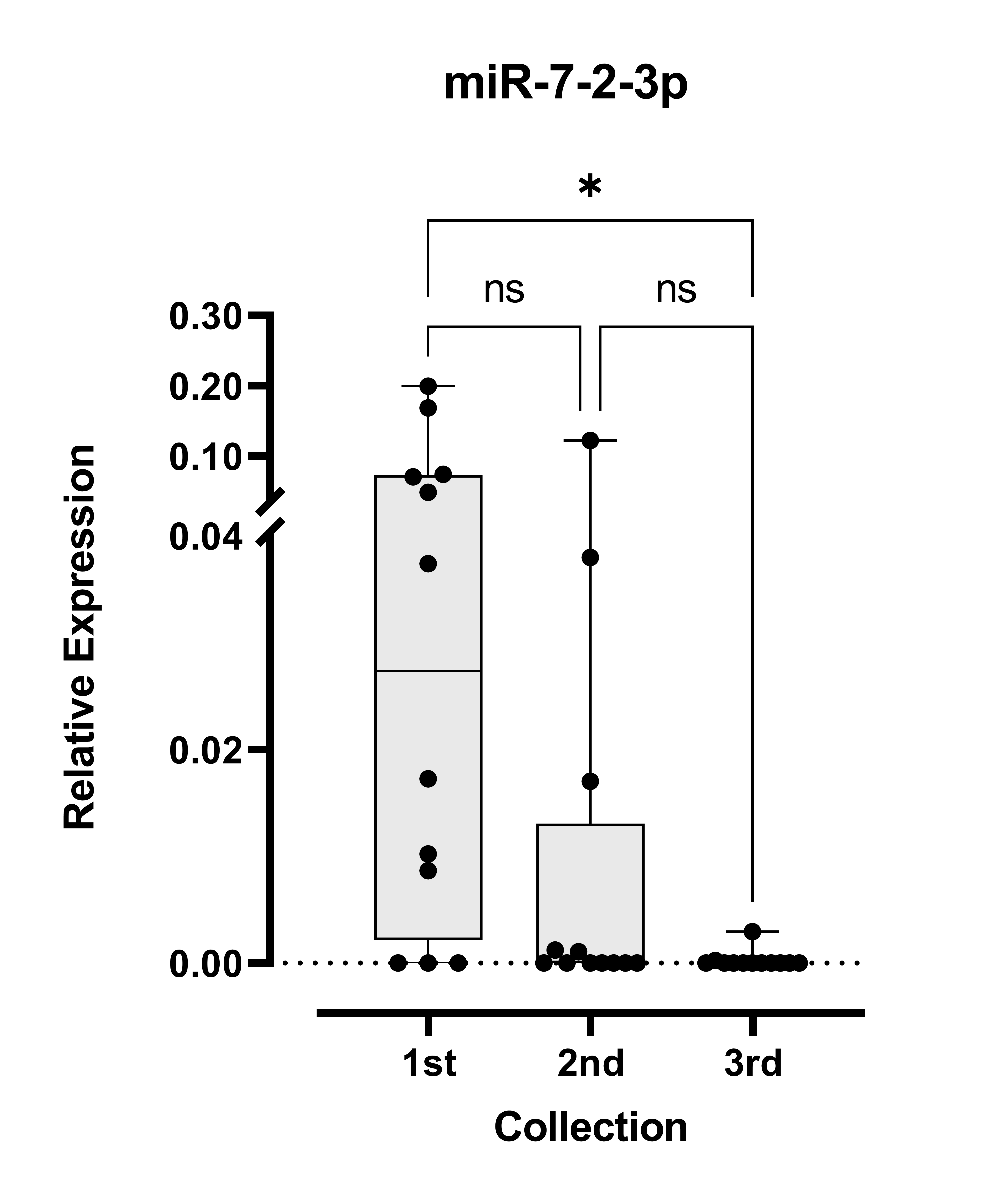

Supplement: Supplementary file 3 — Supplementary Fig. 3 Relative expression of the 8 miRNAs in longitudinal samples of slow progression rate patients. Dots represent mean relative expression values of each sample. Statistical significance calculated using Kruskal-Wallis test and Dunn’s multiple comparisons test. * p value < 0.05 (PNG 257 kb) [file 12035_2023_3520_MOESM3_ESM.png]

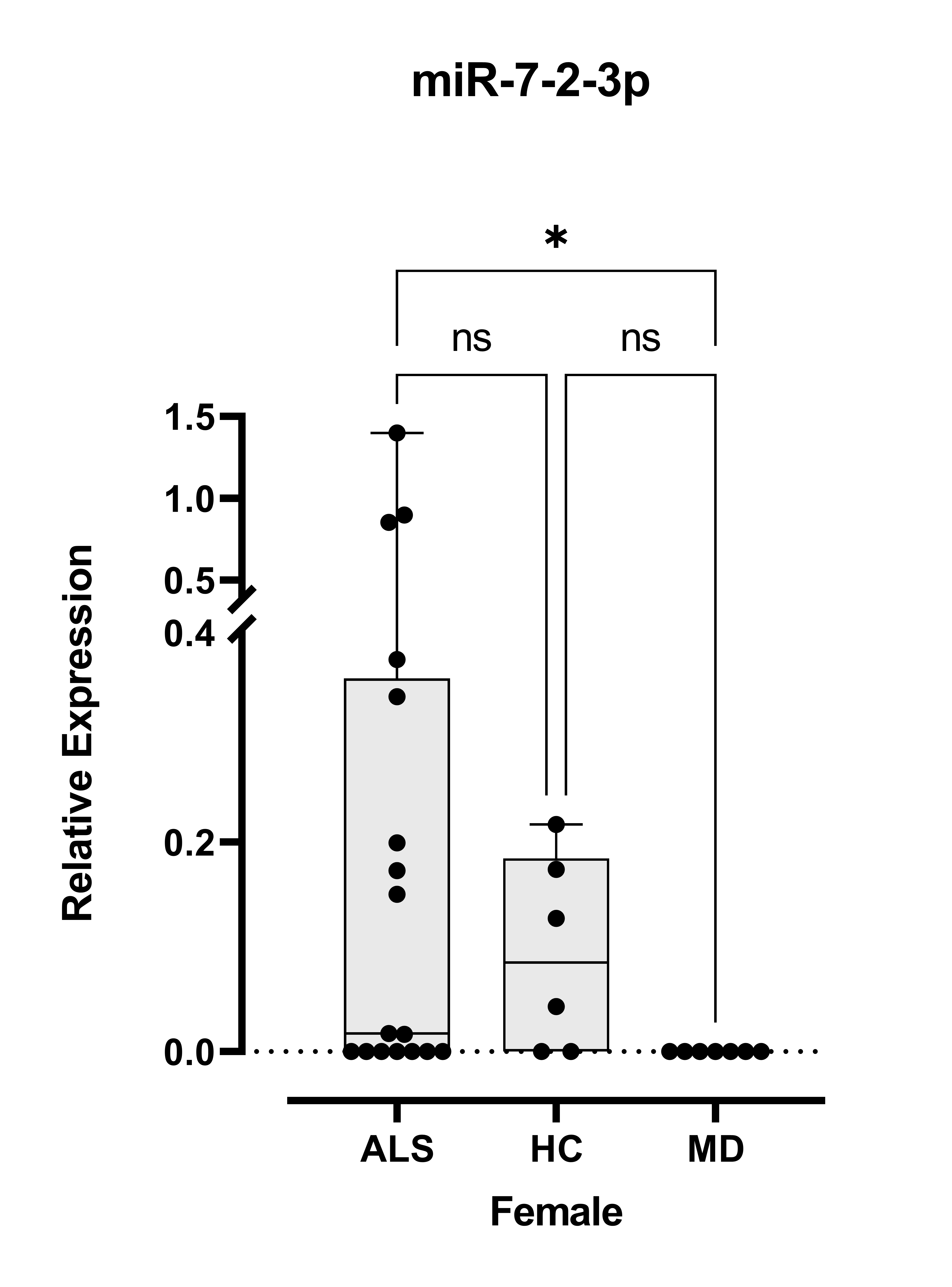

Supplement: Supplementary file 4 — Supplementary Fig. 4 Box and whisker plots of relative expression of miR-7-2-3p considering only females. ALS – N = 17, HC – N = 6 and MD – N = 7. Dots represent mean relative expression values of each sample. Statistical significance calculated using Kruskal-Wallis test and Dunn’s multiple comparisons test. * p value < 0.05. (PNG 249 kb) [file 12035_2023_3520_MOESM4_ESM.png]

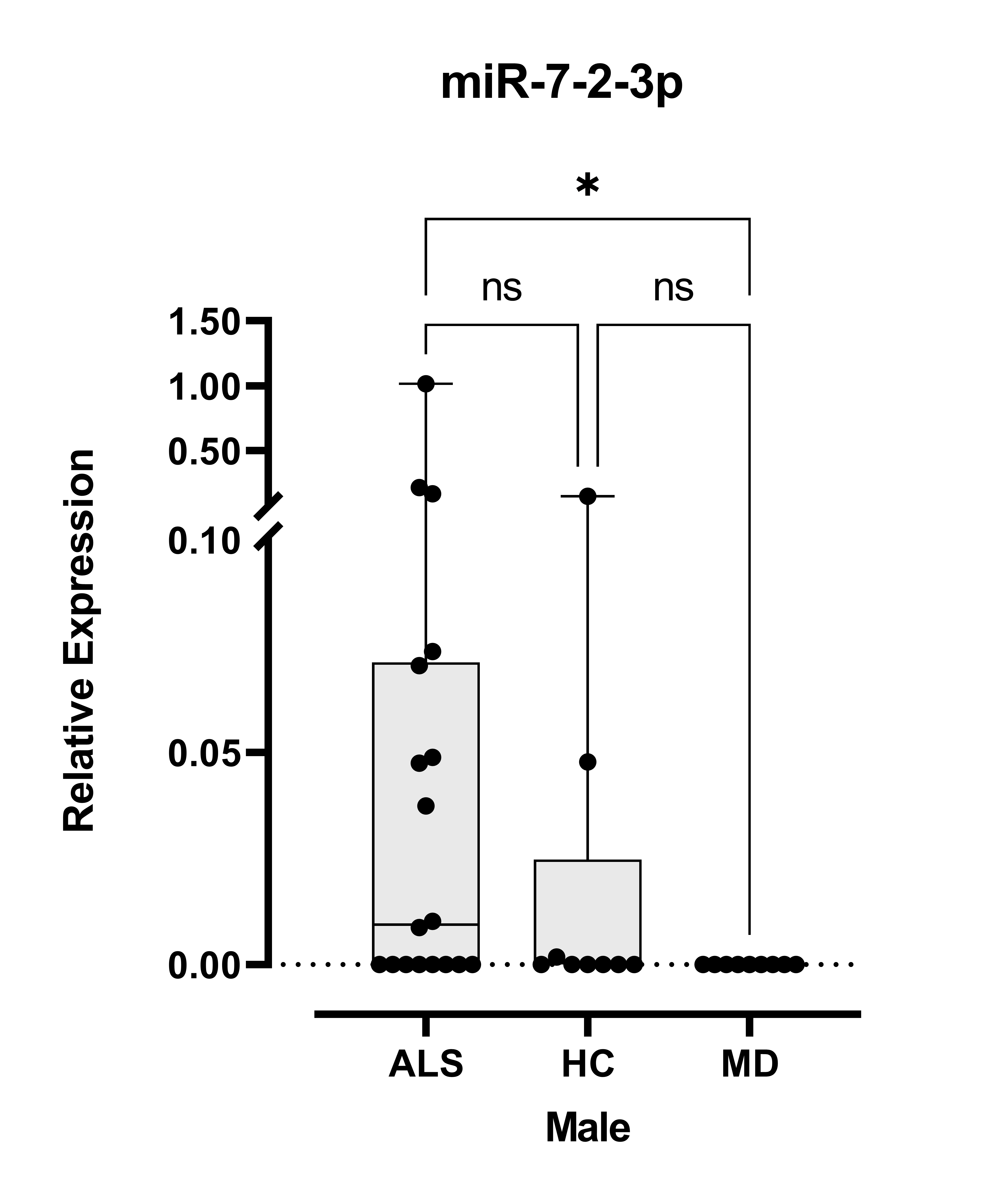

Supplement: Supplementary file 5 — Supplementary Fig. 5 Box and whisker plots of relative expression of miR-7-2-3p considering only males. ALS – N = 17, HC – N = 6 and MD – N = 7. Dots represent mean relative expression values of each sample. Statistical significance calculated using Kruskal-Wallis test and Dunn’s multiple comparisons test. * p value < 0.05. (PNG 246 kb) [file 12035_2023_3520_MOESM5_ESM.png]

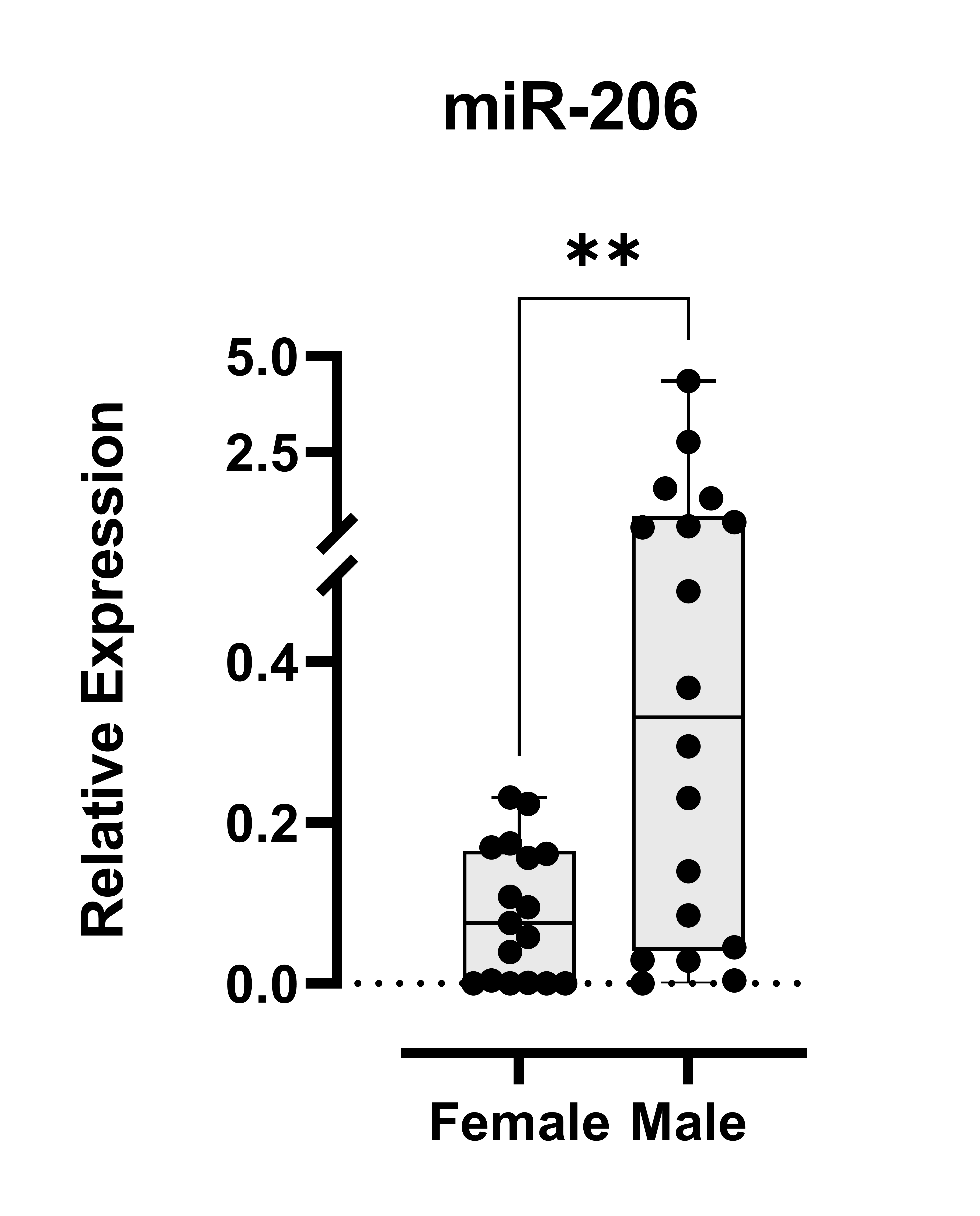

Supplement: Supplementary file 6 — Supplementary Fig. 6 Box and whisker plot of relative expression the statistically differently expressed miRNA detected comparing female and male ALS patients. Female – N = 17, Male – N = 18. Dots represent mean relative expression values of each sample. Statistical significance calculated using Mann-Whitney test. ** p value < 0.01. (PNG 290 kb) [file 12035_2023_3520_MOESM6_ESM.png]
